# Towards an Efficient Method for Large-Scale Wi-SUN-Enabled AMI Network Planning

**DOI:** 10.3390/s22239105

**Published:** 2022-11-23

**Authors:** Marcos Alberto Mochinski, Marina Luísa de Souza Carrasco Vieira, Mauricio Biczkowski, Ivan Jorge Chueiri, Edgar Jamhour, Voldi Costa Zambenedetti, Marcelo Eduardo Pellenz, Fabrício Enembreck

**Affiliations:** 1 Centro de P&I em Sistemas Elétricos Inteligentes (CISEI) — Smart Grid Research Center, Escola Politécnica, Pontifícia Universidade Católica do Paraná (PUCPR), Curitiba 80215-901, Brazil; 2 Programa de Pós-Graduação em Informática (PPGIa), Escola Politécnica, Pontifícia Universidade Católica do Paraná (PUCPR), Curitiba 80215-901, Brazil; 3 SSG — Superintendência de Smart Grid e Projetos Especiais, COPEL Distribuição, Curitiba 81200-240, Brazil

**Keywords:** smart grid communication network, key device positioning, AMI network planning

## Abstract

In a smart grid communication network, positioning key devices (routers and gateways) is an NP-Hard problem as the number of candidate topologies grows exponentially according to the number of poles and smart meters. The different terrain profiles impose distinct communication losses between a smart meter and a key device position. Additionally, the communication topology must consider the position of previously installed distribution automation devices (DAs) to support the power grid remote operation. We introduce the heuristic method AIDA (AI-driven AMI network planning with DA-based information and a link-specific propagation model) to evaluate the connectivity condition between the meters and key devices. It also uses the link-received power calculated for the edges of a Minimum Spanning Tree to propose a simplified multihop analysis. The AIDA method proposes a balance between complexity and efficiency, eliminating the need for empirical terrain characterization. Using a spanning tree to characterize the connectivity topology between meters and routers, we suggest a heuristic approach capable of alleviating complexity and facilitating scalability. In our research, the interest is in proposing a method for positioning communication devices that presents a good trade-off between network coverage and the number of communication devices. The existing literature explores the theme by presenting different techniques for ideal device placement. Still rare are the references that meticulously explore real large-scale scenarios or the communication feasibility between meters and key devices, considering the detailed topography between the devices. The main contributions of this work include: (1) The presentation of an efficient AMI planning method with a large-scale focus; (2) The use of a propagation model that does not depend on an empirical terrain classification; and (3) The use of a heuristic approach based on a spanning tree, capable of evaluating a smaller number of connections and, even so, proposing a topology that uses fewer router and gateway positions compared to an approach that makes general terrain classification. Experiments in four real large-scale scenarios, totaling over 230,000 smart meters, demonstrate that AIDA can efficiently provide high-quality connectivity demanding a reduced number of devices. Additional experiments comparing AIDA’s detailed terrain-based propagation model to the Erceg-SUI Path Loss model suggest that AIDA can reach the smart meter’s coverage with a fewer router positions.

## 1. Introduction

The power grid is a complex system that includes the elements responsible for generating, transmitting, and distributing energy [1]. The authors in [2] explained that existing resources must be optimized through intelligent technologies called smart grids (SGs), which integrate new technologies to improve the monitoring and control of operations, as well as the generation and distribution of energy. Furthermore, it is essential to adapt to variations in demand and facilitate the evolution of energy distribution systems.

In this context, the smart grid concept can significantly improve electrical systems’ robustness and efficiency through an integrated communication network. According to the authors in [3], smart grid communication will improve the quality of power, allowing the integration of different energy sources and enabling the efficient creation of new products and services. In addition, an important difference between traditional and smart grids is the two-way communication capability provided by the SG [4].

In a smart grid, the advanced metering infrastructure (AMI) benefits from bi-directional communication for monitoring and control purposes, enabling reliable and secure high-speed communication between smart meters at the end-user side and the smart grid control center (CC) [4]. This AMI communication is indispensable for the reliable operation of the grid, as explained by [5], which refers to the AMI as a factor for increasing the grid’s reliability.

In an AMI communication network, positioning key devices (routers and gateways) is a complex task. It initially requires knowledge about the positioning of smart meters (SMs), distribution automation (DA) devices, and poles. Knowing the technical information about the communication technology and the geographic area is also important. The planning of an AMI network entails particular concerns regarding the costs and overall performance of the communication network because the adequate positioning of key devices can significantly reduce the total deployment cost. Therefore, when designing an SG communication network, in addition to prioritizing smart meters’ connectivity, we must also take into account the positions of distribution automation (DA) devices. The communication network for managing DA equipment must have high performance and reliability, and thus their connection to the backhaul network is a requirement.

Additionally, a more detailed evaluation of inter-device connections in the planning stage helps to anticipate the occurrence of potentially unreliable links during the implementation phase. In our research, the interest is towards proposing a method for positioning communication devices, presenting a good trade-off between network coverage and the number of communication devices. The existing literature explores the theme by presenting different techniques for ideal device placement. Still, rare are the references that explore real large-scale scenarios or that explore the communication feasibility between meters and key devices meticulously, considering the detailed topography between the devices. Furthermore, we are not aware of device positioning methods that prioritize candidate positions with DAs.

Thus, the main contributions of this work are the following:

- The presentation of an efficient AMI planning method with a large-scale focus: Four real large AMI network scenarios, including urban and rural areas, are used in the experiments to evaluate the method’s performance in large-scale projects. These scenarios include more than 230,000 smart meters. Experimentation with large-scale real data is not common in the existing literature. We believe that exploring large scenarios allows us to evaluate the proposed method under real conditions, verifying the method’s behavior for regions with different concentrations of meters and poles which demand a large coverage area and can present very different terrain characteristics.
- The use of a propagation model that does not depend on empirical terrain classification: A detailed propagation model, including terrain diffraction loss, is applied for the link budget calculation. Instead of using a standard and general link budget approach to compute wireless link losses, the proposed method employs a detailed terrain profile analysis between the smart meters and positions of routers and gateways, leading to a more accurate link quality estimation. An additional experiment compares AIDA (AI-driven AMI network planning with DA-based information and a link-specific propagation model) to the classic Erceg-SUI/IEEE 802.16.3 Suburban Path Loss model [6,7]. The analysis shows that AIDA with its proposed path loss model can propose topologies with fewer routers because it applies a detailed terrain profile in the path loss link analysis. It is rare in the literature research that explores the complete analysis of the topography profile for the path between the devices, mainly because of the number of meters and poles to be evaluated. However, in our proposed method, we make this viable using different strategies (heuristic and grid-based approaches) to minimize the number of connections to be computed.
- The use of a heuristic approach based on a spanning tree and clustering, capable of evaluating a smaller number of connections and resulting in efficient topologies that use fewer routers and gateways: This research proposes a heuristic (AI-driven approach) for planning key devices’ positioning in large-scale AMI wireless networks. The strategy prioritizes using poles with DA devices to enable, whenever possible, the positioning of routers and gateways in locations close to the backhaul network. This applies a grid-based heuristic to determine the candidate positions, minimizing the number of pole positions to be evaluated. In addition, a simplified mechanism for the multihop connectivity analysis based on a minimum spanning tree (MST) heuristic is employed to minimize the number of connections to be analyzed. The selected strategies aim to balance complexity and final solution quality. Different approaches are found in the literature to deal with the gateway positioning problem, some combining different techniques. However, using a grid-based candidate position selection that prioritizes the use of DA device positions, combined with an MST heuristic to explore multihop and minimize the number of connections to be analyzed, is not common.

The remainder of this article is organized as follows: Section 2 describes the architecture of the AMI network and the application scenario. The related work is presented in Section 3. The proposed strategy is detailed in Section 4. Section 5 presents the results of the experiments, where different scenarios are used to evaluate the method’s applicability. Section 6 reviews the research’s main objectives, evaluates the results, and presents our future works regarding the method. Finally, the conclusions are drawn in Section 7.

## 2. Smart Grid Network Architecture

The two main components of a smart grid communication network are the AMI network and the automation network. In both networks, we must establish two-way communication with the control center for data acquisition and management purposes. Using a standardized wireless communication architecture, the AMI network connects smart meters, routers, and gateways. The automation network is mission-critical because it connects DA devices to the smart grid communication infrastructure. The automation network architecture is highly dependent on the correct positioning of the key devices.

### 2.1. General Network Architecture

This study considers the use of the Wi-SUN (Wireless Smart Utility Network) wireless communication standard (Wi-SUN Alliance^®^ [8]). The Wi-SUN standard implements a mesh network architecture based on the IEEE 802.15.4g standard [9], using RPL (IPv6 Routing Protocol for Low Power and Lossy Networks, RFC6550) [10] as a routing protocol at the network layer. The mesh network allows multihop communication between meters, routers, and gateways.

The network planning involves many elements, including smart meters, gateways, routers, poles, and backhaul network components, and it can be classified as an NP-hard problem [11]. Additionally, a set of constraints is associated with the network design, such as ensuring maximum throughput with the lowest possible latency at a reduced cost. The network performance is highly dependent on the placement of key elements for the communication process between the neighborhood area network (NAN) region of the smart grid, the gateways, and the wide-area network (WAN) region, where the CC is installed.

**Figure 1:**
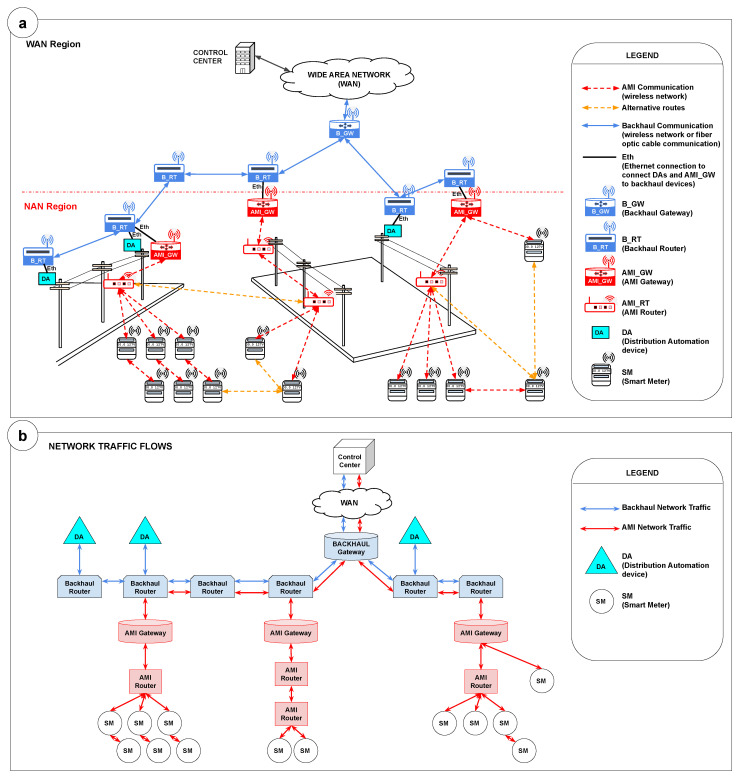
Smart grid scenario. (**a**) Overview of a smart grid scenario, highlighting the main elements in the NAN and WAN regions considered in this research. (**b**) Diagram of Backhaul and AMI network traffic flows to demonstrate the main elements in transferring information between endpoint devices and the control center.
- Regarding the Backhaul network, the elements of interest for this study include the Backhaul Routers, with which the DA devices are connected and which also allow the connection of AMI gateways, and the Backhaul Gateway, which interfaces between the Backhaul network and WAN network for forwarding messages to/from the control center. In this study, when referring to routers and gateways, we are referring in a simplified way to AMI Routers and AMI Gateways.
- The automation and metering communication network infrastructure are based on the existence of poles, as it occurs in many companies worldwide. The advantage of using the poles is based on the fact that they are part of the company’s asset list, minimizing the need to contract third-party infrastructure. In addition, poles offer the supply voltage necessary for the setup and operation of the communication devices and present a favorable height for the positioning of the routers and gateway devices.
- DAs are installed on poles and mainly in the overhead power network. Underground power networks are restricted to small areas in Brazil, in some urban centers, and they are treated as an exception (not in the scope of this study). Communication with DA equipment uses distributed network protocol (DNP3) over an IP using pooling and not unsolicited messages due to a limitation of the supervisory control and data acquisition (SCADA) monitoring system.
- The management of the information flow from the endpoints to the control center considers that data from the AMI elements (e.g., smart meters) and the DAs will share the physical infrastructure of the Backhaul network. However, the information flows through different VLANs and with different priorities, explained as follows: (i) The energy consumption and voltage quality monitoring information from the meters is usually obtained in the AMI network through a pooling mechanism controlled by the control center, which uses an algorithm to make a scheduled pooling to distribute the reading throughout the day and avoid congestion. This algorithm, in general, can control the reading spatially (establishing different regions for the reading) and temporally (to perform the reading of different areas in different periods). An example of a scheduled smart meter reading algorithm is presented by the authors in [12]; (ii) Regarding the DAs (Backhaul network), they are considered high-priority devices; thus, their status is read more frequently (high-frequency reading) as the control center continuously monitors them and acts on them as quickly as required. Despite this high-frequency reading, it is essential to highlight that the number of DAs in a smart grid is considerably inferior to the number of smart meters. Thus, their traffic represents a high frequency of readings but for a small number of elements.
- Finally, the AMI network function is not restricted to metering and billing. It comprises bidirectional communication that allows a remote to switch off/reconnect consumers’ houses’ energy—in addition to supporting “last gasp” alarms informing the lack of energy in consumers’ houses and being able to map the defective sections and coordinate maintenance teams with greater assertiveness. The DAs communication network (wireless backhaul network) is provided by a backup energy system (batteries) to enable maneuvers even during shutdowns.

Figure 1a presents an example of a smart grid scenario. Wireless communication between the elements of the NAN region takes place according to the messaging protocol used by the network. In this study, we consider using the RPL protocol, which allows the existence of different routes intending to minimize the points of failure using alternative parent nodes (backup nodes) for forwarding messages. Analyzing from the point of view of each meter, different communication options are possible, either by connecting the meter directly to a gateway, a router, or even using multihop forwarding messages through other meters.

The smart meters, routers, gateways, and DA devices are in the NAN region. DA devices include voltage regulators, and automatic reclosers, among other equipment. All these elements must be connected to ensure communication with the backhaul network, which connects the main communication elements and establishes a reliable two-way access channel from NAN to the CC of the smart grid.

In a typical smart grid scenario, the correct positioning of communication key elements, such as gateways and routers, assures communication between a large number of SMs and the CC and between the CC and DA devices. In addition, it is essential to highlight that, usually, these elements (smart meters and DAs) are dispersed over a large geographic area, introducing complexity to the positioning planning.

In practice, gateways and routers are installed on poles. The installation is usually done in areas with a high concentration of meters and equipment to be connected. A set of candidate positions can be established from the set of poles. For the AMI planning and key devices positioning, special preference must be given to the use of poles hosting DAs, as they are usually already installed in the region and because of their importance in the electrical infrastructure. In some cases, the DAs may already be interconnected via fiber optic cable, thus reinforcing the use of these resources on a preferential basis.

### 2.2. Network Planning Constraints

The constraints presented in this section are based on directives for the implementation and operation of the smart grid of a large electric power company in the state of Paraná, in southern Brazil. The constraint list includes:

- The smart grid structure comprises two wireless networks (Figure 1b): the Backhaul network and the AMI wireless network based on Wi-SUN technology. In addition, the backhaul network is connected to an optical network (WAN backbone) at electric substations.
- The wireless backhaul network is segmented into three virtual local area networks (VLANs) with different traffic priorities. The first VLAN is for radio monitoring and has the highest priority. The second VLAN is for the equipment automation of the distribution power network and has the second highest priority. Finally, the third VLAN is for the AMI communication traffic. This VLAN transports smart metering data traffic from the Wi-SUN network and has the lowest priority. The AMI and DA communication networks are separated by VLANs at each trunking point with the physical network (substation, VHF stations, or branch) as this increases the security level of the communication network as a whole.
- The main elements of interest in the AMI network topology for this research include (i) smart meters, which measure energy consumption; (ii) AMI routers, with which the meters connect and which are responsible for forwarding messages through the network; and (iii) AMI gateways that accept connections from routers as well as direct connections from meters and that, in addition to relaying messages, serves as a communication interface between the AMI network and the Backhaul network.

## 3. Related Work

In this section, we present different AMI planning strategies for key device positioning found in the literature. They relate to the smart grid scenario and explore diverse techniques to define the ideal placement of gateway devices in the communication network.

### 3.1. Approaches for Key Devices Positioning

The state-of-the-art literature explores many strategies for positioning routers and gateways in the smart grid wireless communication scenario. In this section, we analyze different selected references, especially to identify the authors’ research context and the algorithmic approach explored to deal with the positioning problem.

The authors in [13] propose an algorithm to determine the optimal location of concentrators in smart grids, based on the ZigBee Mesh IEEE 802.15.4 communication protocol in the final access and Global Packet Radio Service (GPRS) communication in the concentrator of the mesh network, directly connecting to the utility’s head–end system (backhaul). They assume a smart grid architecture with neighborhood area network (NAN) in which the meters are located, wide area network (WAN) where the collectors are positioned (usually installed on street light poles), and including a head–end system, the layer in which the network management is performed. Then, they present a proposal for a methodology for positioning concentrators in a Mesh network (in this case, a ZigBee network) based on the position of poles available in the region, aiming to optimize the network’s performance. Then, all available poles become candidate positions for the concentrator installation. Additionally, they set a goal to find the minimum hops between meters and concentrators, aiming to find the shortest path that minimizes the cost of routes. Furthermore, they use different algorithms to establish the best route between two points. In this case, they use Dijkstra, Bellman–Ford, and BFS (Breadth-First Search). In situations where they define that more than one concentrator must be positioned, they use k-means to form clusters, having candidate positions (poles) as centroids. The proposed algorithm includes the following steps: obtaining the geographic coordinates of the meters and poles; if more than one concentrator must be positioned, they suggest the use of clustering (k-means) to define the centroid’s position; the next step is choosing the position for the concentrator on one of the candidate poles; applying a shortest path algorithm (BFS, Dijkstra, and Bellman–Ford); evaluating the number of hops; changing the position of the concentrator; choosing as the final position the one with the least number of hops.

In [14], the authors used the K-means-Dijkstra approach proposed by [13] to minimize the number of hops in the network. Additionally, they explored the recursive algorithm proposed by [11] to optimize the positioning of concentrators in smart grids aiming, among other factors, to balance the number of meters connected to the concentrators (load balancing) and the maximum number of hops.

According to the authors in [11], in a network with many hops, a significant delay at each hop can occur due to wireless channel contention, packet processing, and queuing. They clarify that the delay depends on the number of hops between the source and the gateway. Thus, to minimize the delay, it is necessary to determine a cluster radius or maximum depth of the tree that interconnects the communication network nodes (rooted in the gateway) to ensure the quality criteria. To establish the position of gateways in wireless mesh networks, they divide the set of nodes formed by the meters into disjoint clusters. One node acts as a gateway in these clusters with the other nodes connected to it. A gateway-rooted spanning tree is used to aggregate and secure message traffic/forwarding in each cluster. The proposed algorithm is based on the dominant set (DS) concept, using recursive approximations of the minimum DS problem. The algorithm uses an adjacency matrix to represent the connectivity graph between network nodes. They consider nodes that are one hop from each other as adjacent, and they use a greedy algorithm to select a node *v* that will be the centroid of a cluster. Then, the algorithm assembles an additional set with the candidate centroid and its neighbor nodes and analyzes whether the cluster is viable. A cluster is considered viable if a spanning tree, rooted in *v* and covering all cluster nodes, satisfies the cluster size and relay load constraints. It continues the iteration (recursion) as long as there are nodes to be clustered. The recursive algorithm’s stopping condition occurs when the next iteration’s cluster radius exceeds a specific maximum radius *R*.

The authors in [15] highlight that, although many studies address the problem of the optimization of device placement in wireless networks, few specifically address the location of concentrators in AMI networks. Compared to the work by [13], they also consider existing poles as candidate positions for installing concentrators. They aim to establish the concentrator position that minimizes the number of hops, maximizes throughput, and keeps delay within certain limits. To analyze the average throughput and delay, they use an M/M/1/K queue model and present a set of formulas for calculating throughput. This includes packet loss probability, service rate, time-of-service with no errors, the packet error rate at the physical layer, and traffic at a given node. For the delay analysis, they consider the queue size, the waiting time, the end-to-end delay, and the end-to-end average delay. A similar study is presented by [16], proposing a channel-aware optimal location approach for the data concentrator unit placement in smart grids.

For the positioning of concentrators in problems of smart grid networks expansion, the authors in [17] used mixed-integer non-linear programming (MINLP) and mixed-integer linear programming approaches (MILP) to minimize network congestion by optimizing residual buffer capacity by positioning data concentrators and network routing. They proposed an approach for adding new devices to AMI networks that are already in operation, maximizing the quality of service (QoS) performance for customers, providing redundant connectivity in cases of security threat or interruption in power supply, and assisting in network expansion. They suggest using the path loss propagation model to identify the communication radius around data concentrators and analyze different restrictions to establish the best position for this type of equipment.

The authors in [18] proposed using clustering techniques for the problem of data aggregation points (DAPs) positioning in NAN to minimize the distance between DAPs and SMs, dividing the neighborhoods into subnetworks. With this, they also seek to reduce the number of hops to up to 3 hops. Furthermore, they introduced a new metric called coverage density, which defines whether the planning done for a given zone ensures the necessary coverage. The authors cite that the problem of positioning DAPs is an underexplored topic in the domain of smart grids. The methods used by the authors include Haversine distance, Floyd–Warshall (FW) algorithm to find the (shortest path) route between a given node and another node in the network, and the k-Medoids clustering algorithm, which takes into account the distance between smart meters and the transmission range of the meters.

Minimizing the distance between data aggregation points (DAPs) and smart meters served by them can be the requirement of a device placement problem. About this, the authors in [19] used a network partitioning approach, using a clustering algorithm clustering-based DAP placement algorithm (CDPA) that performs the clustering aiming to minimize distance. The authors also used the Floyd–Warshall algorithm for the shortest path search and the distance between nodes as information considered by the method.

In [20], the authors discussed the positioning of access points (APs) in smart grids with a communication network implemented with a power-line communication (PLC) network. First, they establish an optimization model for the location of the AP that minimizes the cost of installing APs, while satisfying the constraints of reliability, network delay, and resiliency. Then, they propose an improved genetic algorithm (GA) to solve the optimization problem. As for the aspects of design constraints, they mention (a) the minimization of the cost of building the network, mainly the costs of installing the APs, (b) the maximization of the average level of reliability under normal operating conditions, (c) to ensure that the ENs (end-nodes) are always connected to at least one AP to keep the network running at a proper level even if a link becomes unavailable (i.e., ensuring that the network reliability remains above a predefined threshold), and (d) reduction in the communication delay to meet application requirements. The algorithm used to solve the planning problem is an improved GA. The density function is introduced based on the standard GA to avoid the local optimum and maintain population diversity.

The analysis of the topologies of a power grid and the communication network can be done together, as demonstrated by the authors in [21]. This study has as its main objectives to establish the coordinates of the DAPs and to minimize the total average delay of the system, considering the volume of data traffic and the minimization of costs. They aim to do this without compromising QoS. This is achieved by focusing on the idea of aggregating and compressing the data associated with the same power feed in the appropriate DAP before being sent to the utility center (UC). The authors point out that having smart meters fed by different feeders of the energy network, connecting to the same DAP in the communication network, can lead to inefficient data aggregation. Therefore, the problem of positioning DAPs should not consider the communication network isolated from the electrical network. The authors formulate the problem as a mixed-integer non-linear optimization problem, and the optimization is done with a genetic algorithm.

Network latency minimization is a common need in different studies and is addressed by [22]. The authors formulated a DAP problem and then used a clustering approach to network partitioning to minimize the maximum latency of data propagation between each DAP and the associated meters. They used a Dijkstra algorithm to calculate the shortest path between two nodes and consider the Haversine distance between the elements. Then, they used a clustering-based DAP placement (CDP) approach. The main network characteristics the authors considered include the number of meters, the position of meters, the transmission range, and the number of DAPs.

Gateway positioning taking energy efficiency into account is explored by the authors in [23]. For the gateway positioning, they use an optimization method mainly based on distance (Euclidean and Manhattan), also considering the distance between coordinating devices (CDs) and gateways, the throughput, power consumption, load balancing, and link capacity. The method is performed in two stages: in the first stage, the candidate position selection is made using the Euclidean Distance, and in the second stage, the Gateway location selection is made using the Manhattan distance.

The positioning of gateways to increase the capacity of the backhaul network by minimizing the average number of hops (ANH) is addressed by the authors in [24]. The study has applications in 5G ultra-dense networks but is included in our analysis because of our interest in controlling the number of hops. The methods used by the authors include clustering (using k-means and k-medoids algorithms) and the Dijkstra algorithm, used to find the average number of hops and to associate small cells with gateways (by identifying the shortest path). The network characteristics evaluated by the proposed method include the number of hops, the throughput, and the number of simultaneous transmissions.

The concept of gateway node placement problem (GNP) was explored by [25] to establish the smallest possible number of gateways to satisfy QoS requirements in search-and-rescue environments in a wireless mesh network (WMN). The authors treat the gateway placement problem (GNP) in combination with the router placement problem (RNP). The techniques considered by the authors include clustering strategies, area decomposition, and a heuristic approach (heuristic graph clustering technique). The method proposed by the authors is performed in two steps: the first step of the algorithm ensures that the calculated placements of the router nodes for a given deployment region meet the objectives of the RNP problem and its constraints. This means that the resulting WMN backbone network configuration maximizes the network coverage while maintaining network connectivity and minimizes the number of router nodes (RNs) used. The second step (GNP) ensures that the number of assigned gateway nodes is minimal and the division of the network topology graph into a set of disjoint clusters (subnets) satisfies three QoS constraints: RQoS (maximum communication delay), LQoS (maximum relay load for each RN) and SQoS (gateway throughput). The authors proposed the RRT-WMN algorithm (where RRT = rapidly exploring random trees) and used it combined with a heuristic approach to graph clustering. They used the RRT-WMN algorithm to resolve router placement. Then, the resulting network topology graph, along with the QoS constraints, is used as input to a graph clustering approach (which integrates the Weighted Recursive Dominating Set algorithm). Among the characteristics considered by the methods are obstacles, signal range, delay, load on routers, and throughput/gateway capacity. The authors highlight that an important measure of WMN network performance is network connectivity, which quantifies how well the routers are interconnected. They indicate that connectivity is even more important than network coverage or customer coverage, as it ensures that the router nodes are interconnected. In short, the GNP problem is about finding a minimum number of GNs and their placements to ensure a sufficient level of QoS based on criteria that directly influence network performance measures, such as communication delay, router load, and the capacity limits of GNs.

Throughput optimization is the objective of the study by [26], which addresses gateway placement in WMN. They consider the number of gateways to be placed and the interference model of the network. The method proposed by the authors can be extended to multi-channel and multi-radio mesh networks. The authors proposed the positioning based on a grid, evaluated different positions for the gateways, and selected the combination that ensures the highest throughput. The proposed strategy is compared with random placement and with fixed placement. According to the authors, the grid-based positioning showed the best result in the experiments performed. Regarding the techniques, the authors use mixed-integer linear programming for the optimization/maximization of throughput (routing problem), a greedy algorithm for interference-free link scheduling, and a grid-based gateway placement scheme (which uses the linear programming method used for throughput as an assessment tool) for position selection. For the positioning processing, the main information considered by the method includes the analysis of: range (for interference range analysis), achieved flow (relation between the flow obtained concerning the flow demanded, treated as a constraint), and the total scheduled traffic.

Meta-heuristic approaches are explored by [27] for the positioning of gateways in WMN. For this, they use Genetic Algorithm (GA) and Simulated Annealing (SA), considering the number of gateways and the number of hops that packets need to travel between the source and the destination (router/gateway). They aimed to minimize the variation of hops between routers and gateways (VAR-MR-IG-Hop) of the routers (MR, Mesh Routers) to ensure that the gateways are properly positioned. In addition, the authors used the Dijkstra algorithm to calculate the shortest path between each router and all gateways in the network.

The authors in [28] discussed gateway positioning based on graph clustering and the use of a repairing genetic algorithm (RGA) to work with such graphs, to repair unfeasible solutions. RGA differs from GA by detecting and repairing unfeasible solutions generated by crossover and mutation operations, in addition to being computationally efficient, with reduced processing time compared to GA.

### 3.2. Comparative of Key Devices’ Positioning Approaches

In this section, we compare our study to the characteristics of selected references which explore the key device positioning problem to highlight the main points of our research.

Table 1 aimed to present the comparison of the main characteristics of the method proposed by this research to different approaches used by selected references found in the literature that explore the gateway/router positioning problem. In addition, Table 1 highlights some of the innovative features of the method, such as its detailed topographic profile analysis, the consideration of DA positions, and its experimentation with real large-scale data.

Regarding the techniques applied by different references to solve the key devices positioning problem (see Table 1), the use of heuristic algorithms [13,29,30,31,32,33] and clustering strategies [13,16,18,21,22,30,31,32,34] is common. This problem is generally classified as an optimization problem because of the number of equipment, connection possibilities, constraints, and objectives. Regarding the problem formulation, some authors cite that this problem can be modeled as a linear/non-linear programming problem, as expressed by [17,21,29,30,32]. In addition, some authors classify the problem as a set covering problem [29,31,33], or as a facility location problem [17,18,34]. Metaheuristic approaches, including genetic algorithms, are also explored [21,34], demonstrating that various strategies can be used to solve the problem.

Despite experiments considering real scenarios, it is common to observe simulations using small datasets [13,16,18,22,33], or small synthetic datasets as in [17,21,35]. However, large real scenarios with more than 230,000 smart meters are used in our experiments to evaluate our proposed method, checking whether the average received power values calculated for the links are within the established threshold.

Most strategies presented in Table 1 employ simplified propagation models to evaluate the potential wireless connection between the devices during the planning process. Usually, they do not consider terrain-specific information to estimate the signal loss from an SM to another coordinate (another SM or a candidate position) but assume a general scenario for the region, classifying it as hilly or flat, with moderate or heavy vegetation, or indicating that the region is an urban, suburban, or rural area. Some of them model the planning process as a simple distance-based clustering problem. In practice, some deployment scenarios can be challenging by presenting dense and sparse regions with irregular terrain profiles in suburban, urban, or dense urban scenarios. In these cases, despite demanding greater computational complexity to be solved, the optimal placement of devices can effectively reduce deployment costs.

**Table 1:** Selected references about key devices positioning in SG.

	References													
	**This Study**	**[22]**	**[21]**	**[18]**	**[35]**	**[29]**	**[30]**	**[13]**	**[31]**	**[32]**	**[33]**	**[16]**	**[34]**	**[17]**
Heuristics approach	√					√	√	√	√	√	√			
Metaheuristics approach			√										√	
Network partitioning approach		√		√										
Clustering-based approach	√	√	√	√			√	√	√	√		√	√	
Linear/non-linear programming modeling			√			√	√			√				√
Set covering problem						√			√		√			
Facility location problem				√									√	√
Routeing assignment problem														√
Analytical model					√									
Propagation model w/detailed terrain profile	√													
Propagation model w/simplified terrain profile			√			√	√		√	√		√	√	√
Poles as candidate positions	√					√	√	√		√	√	√		√
Prioritize poles with DA devices	√													
No. of SMs (experiment w/real data)	234,797	294		891		29,002		67			381	31		
No. of SMs (experiment w/synthetic data)			348		81	N.A. *	17,121		24,011	8020			5000	275

* N.A.—not available.

Regarding the use of general propagation models to evaluate the path/diffraction loss in an SG key device positioning problem, the authors in [17] present a work that explores the placement of data concentrators for the expansion of smart grid communication networks and use the Stanford University Interim (SUI) propagation model [7] to classify the terrain. They select the most appropriate terrain type to evaluate path loss and establish the communication range. According to [17], the SUI model presents the following types of terrains: Category A (maximum path loss)—mountainous terrain with moderate-to-heavy vegetation; Category B—flat terrain with moderate-to-heavy tree densities, or mountainous terrains with light tree densities; and Category C (minimum path loss)—flat terrain and light tree densities.

The authors in [29] evaluated the link quality between SMs and poles using the link successful delivery rate (SDR). They applied the general extended Hata-SRD path loss model presented in [36] which only considered three scenario classifications: rural, suburban, and urban. In contrast, our study estimated the connectivity of an SM and a candidate position using a more detailed approach. We employed a path loss model, which includes link-specific terrain profile information to compute diffraction losses. This allows a better estimate of the average link received power for the complete path between each SM and the CPs around it. In addition, we prioritize the use of poles with DAs for cost and performance reasons, according to the constraints presented in Section 2.2.

### 3.3. Comments

Analyzing existing references in the literature on the key device positioning problem helps to identify the different strategies evaluated by different authors in their research, the difficulties encountered, and the successes achieved with the experiments. Each study has its original purpose of proposing a method or approach that best helps solve the positioning problem in different contexts. They may contain particularities specific to the study’s region or the network technology used when the survey was developed.

The consulted references include recent works and older research chosen by their relevance in the area. From them, it is possible to extract insights capable of helping in developing new works, shortening distances, and, in a way, contributing to the creation of new research that will be part of the so-called state of the art. Concepts such as candidate positions, clustering and network partitioning approaches, techniques for finding the shortest path in graphs, or the use of a grid to minimize the number of candidate positions to be evaluated were identified in the consulted references and helped in the building of strategies for the development of our research.

Among the positioning strategies, heuristic approaches are evidenced in several studies. It is motivated by the complexity of the scenario involved (either by the number of devices involved or by the high set of constraints associated with the problem) and by the recognized fact that such strategies, if well implemented, ensure a final solution capable of meeting all established requirements.

Based on this, among the points that we defined as strategic to be explored by our research, we included: (a) The experimentation of large-scale scenarios because we identified many studies using reduced experimentation datasets; (b) A detailed assessment of the topographical profile of the terrain for the calculation of losses, as we identify the existence of works that either consider only the distance between elements, or path loss models that use a general classification for the terrain under analysis; (c) Prioritization of the use of pole positions that contain DAs installed, with the objective of stimulating the positioning of key devices of the AMI network (in this case, routers and gateways) at points that will be mandatorily served by communication elements of the Backhaul network (in this case, positions that should provide for the installation of routers of the backhaul network) and, therefore, minimize the number of devices to be installed, since both automation equipment (DAs) and key devices of the AMI network can share the same equipment.

Finally, proposing a heuristic strategy for positioning aims to generate a method that is easy to understand, capable of meeting the requirements of the problem, and obtaining a solution with received power quality within established limits. Therefore, heuristics are proper even for large-scale problems representing real smart grid scenarios.

## 4. Proposed Method

This study presents the method AI-driven AMI network planning with DA-based information and a link-specific propagation model (AIDA) created for positioning gateways and routers in the smart grid communication network, considering the position of poles and DA equipment.

We consider the average link received power (LRP) for connectivity analysis. The LRP is computed using a detailed propagation model, including diffraction loss. The diffraction loss takes into account the topographic profile between the geographic coordinates of the points in the link budget analysis. The Delta-Bullington method [37] is used to calculate the diffraction loss using the characteristics of the terrain profile between the coordinates. The method estimates the LRP based on the technical characteristics of the devices, including transmission power, receiver sensitivity, and antenna gains.

Regarding AI techniques, the proposed method explores unsupervised machine learning algorithms (clustering algorithms) and graph-processing algorithms (e.g., minimum spanning tree). Furthermore, exhaustive search and greedy search algorithms are used.

### 4.1. Multi-Objective Function Optimization Problem

The method is characterized as a multi-objective function optimization problem and aims to reach the six objective functions described in this subsection. The first objective function aims to maximize the *LRP* value obtained for a connection between a smart meter $v_{i}$ and a key device position $k_{j}$ (1):

(1) $$\mathrm{maximize}\;LRP_{v_{i}\to k_{j}},\;\;\forall \;\;v_{i}\in \left\{V\right\},\;k_{j}\in \left\{K\right\}$$

where $V=\{v_{1},\ldots ,v_{u}\}$ is the set of smart meters and K is the set of key devices positioned. To reduce the installation costs, the second objective function aims to minimize the number of key devices positioned (2).

(2) minimize|K|

where K will be positioned in a subset of the set of candidate positions $C=\{c_{1},\ldots ,c_{z}\}$. Considering the key devices positioned, the third objective function aims to maximize the number of key devices installed in poles with distribution automation, *DA*, equipment (3):

(3) $$\mathrm{maximize}\;|K_{DA}|$$

where $K_{DA}\subseteq K$. Positioning a key device on poles with DA minimizes the setup cost, as it ensures that the device will be installed at a point where the backhaul network has already been configured. The fourth objective function maximizes the number of smart meters connected to a key device to construct the set of connected SMs and to optimize the use of each key device (4):

(4) $$\mathrm{maximize}\;|V_{con_{k_{j}}}|$$

where $V_{con_{k_{j}}}$ is equal to the number of smart meters $v_{i}\in \left\{V\right\}$ connected to a key device $k_{j}\in \left\{K\right\}$. The fifth objective function maximizes the average *LRP* value for the solution (5):

(5) $$\mathrm{maximize}\;\left(\frac{1}{n}\sum_{i=1}^{n}LRP_{v_{i}\to k_{j}}\right),\;\;\forall \;\;v_{i}\in \left\{V\right\},\;k_{j}\in \left\{K\right\}$$

where *n* is the number of connected smart meters. The last objective function minimizes the percentage of unconnected smart meters, $P_{u}$, evaluated at each iteration of the method by the stopping criteria (6). The maximum percentage must be adjusted according to project requirements, and it is set as a parameter for the method execution:

(6) $$\mathrm{minimize}\;P_{u},\;\;\forall \;\;v_{i}\in \left\{V\right\},\;k_{j}\in \left\{K\right\}$$

where $P_{u}\leq P_{u}^{max}$, and $P_{u}^{max}$ represents the stopping criteria.

The placement of routers and gateways is considered an NP-hard problem, characterized by multiple objective functions, demanding complex resolution. The use of an AI-driven heuristic approach (as proposed by this study) aims to reduce its complexity and reach a solution for the problem in a reasonable processing time. Therefore, it is useful in planning large-scale scenarios.

### 4.2. Candidate Positions

In the proposed strategy, we define candidate positions (CPs) as pole positions of the electric power distribution network, including poles with distribution automation equipment attached to them. They are called candidates because their promotion to a router or gateway position will depend on the existence of SMs connected to their position at the end of the planning process.

First, the method prioritizes the selection of poles with DA devices, as these equipment need to be directly connected to the backhaul’s communication network. After that, regular poles are considered to connect the remaining smart meters.

Using poles as candidate positions is justified because these elements are part of the energy distribution company’s asset list and can be easily configured to meet the technical requirements for installing routers and gateways.

Considering the irregularity of the regions’ terrain and the high number of existing poles in each city, the AIDA method uses a grid approach to make an optimized selection of a subset of poles to minimize the computational effort required to choose the most ideal coordinates for positioning the key devices. More details about this process are presented in Section 4.4.3.

### 4.3. Link Received Power

The link received power (LRP) is used as the metric to establish the connection between a smart meter and a candidate position. The LRP is computed based on the transmission power, antenna gains, and channel path loss model. To estimate the link power loss (LPL), we consider the link path loss and diffraction Loss.

The LPL considers the detailed terrain profile, which is constructed as follows: initially, the coordinates and length of the path between the smart meter and the CP are identified; the path is divided into 100 equidistant points, and the terrain elevation at each point is obtained; with the elevation measurements and the position of each point, the detailed profile of the terrain is obtained.

The use of a detailed terrain profile analysis avoids (or minimizes) the need for empirical terrain classification, as it is very difficult (or imprecise) to define whether, for a specific region, the terrain is, e.g., totally hilly or flat, or just 50% hilly with light or heavy tree density, and so on.

The International Telecommunication Union (ITU) (https://www.itu.int/ (accessed on 8 May 2022)) presents models for determining the diffraction losses of radio links in its recommendations. The Delta-Bullington method is presented in ITU-R P.526-13 [37]. It aims to determine the diffraction of a radio link considering the multiple obstacles determined by the terrain profile between the transmitter and receiver points. The Bullington diffraction loss for the path is given by (7):

(7) $$L_{b}^{\mathrm{dB}}=L_{uc}^{\mathrm{dB}}+\left(1-e^{-L_{uc}^{\mathrm{dB}}/6}\right)\times \left(10+0.02\times d\right)$$

where $L_{uc}^{\mathrm{dB}}$ is the knife-edge loss for the Bullington point, and *d* is the distance (in km) between the transmitter and the receiver. The model includes three types of diffraction loss (DL) (more details in [37]):

- Bullington DL for the actual path profile ($L_{\mathrm{ba}}^{\mathrm{dB}}$): For the calculation of $L_{\mathrm{ba}}^{\mathrm{dB}}$, the Bullington method is applied using Equation (7) considering the actual terrain profile with all its elevations. The obstacle that causes the greatest diffraction is considered for the calculation.
- Bullington DL for a smooth path profile ($L_{\mathrm{bs}}^{\mathrm{dB}}$): This diffraction loss considers a terrain without elevations. For the calculation of $L_{\mathrm{bs}}^{\mathrm{dB}}$, the Bullington method is applied using Equation (7) considering an equivalent obstacle with equivalent heights of the transmitter and receiver antennas.
- Spherical-Earth Diffraction Loss ($L_{\mathrm{sph}}^{\mathrm{dB}}$): This diffraction loss takes into account the Earth curvature and it is calculated as the interpolated diffraction loss, given by (8):
  (8) $$L_{\mathrm{sph}}^{\mathrm{dB}}=\left[1-h/h_{\mathrm{req}}\right]A_{h}$$
  where h is the smallest clearance height between the curved-earth path and the ray between the antennas, $h_{\mathrm{req}}$ is the required clearance for zero diffraction loss, and $A_{h}$ is the diffraction loss for the path using the modified Earth radius. If $A_{h}$ is negative, the diffraction loss for the path is zero, and no further calculation is necessary.

The link diffraction loss (*LDL*) for the general path is:

(9) $$LDL\;\left(\mathrm{dB}\right)=L_{ba}^{\mathrm{dB}}+\max\left(L_{sph}^{\mathrm{dB}}-L_{bs}^{\mathrm{dB}},0\right).$$

The path loss (*PL*) corresponds to the reduction in power density of a radio wave as it propagates through the channel [38]. This signal attenuation is usually the result of physical propagation phenomena such as reflection, refraction, diffraction, and scattering [39]. Considering the radios’ operating frequency, the diffraction phenomenon is particularly relevant for precise path loss estimation. For the method proposed in this study, *PL* is calculated considering the log-distance path loss model defined in Equation (10), where λ is the wavelength, and *d* is the distance between transmitter and receiver, in meters. The parameter β is the path loss exponent and $d_{0}$ is the reference distance.

(10) $$PL\;\left(\mathrm{dB}\right)=10\cdot \log_{10}\left[{\left(\frac{4\pi d_{0}}{\lambda }\right)}^{2}\right]+10\cdot \beta \cdot \log_{10}\left(\frac{d}{d_{0}}\right)$$

The link power loss (*LPL*) is given by Equation (11).

(11) LPL(dB)=PL(dB)+LDL(dB)

Finally, the link received power (*LRP*) between a smart meter (*SM*) and a router (*RT*) and/or gateway (*GW*) is computed using (12):

(12) $$\begin{array}{cc} LRP\;(\mathrm{dBm})= & P_{tx}^{SM}\;\left(\mathrm{dBm}\right)+G_{tx}^{SM}\;\left(\mathrm{dBi}\right)\\ \; & +G_{rx}^{RT/GW}\;\left(\mathrm{dBi}\right)-LPL\;\left(\mathrm{dB}\right) \end{array}$$

where $P_{tx}^{SM}$ is the SM transmission power, $G_{tx}^{SM}$ is the SM antenna gain, and $G_{rx}^{RT/GW}$ is the router/gateway antenna gain. Equation (12) is also used to calculate the LRP for the link between two smart meters.

### 4.4. AIDA Method

**Figure 2:**
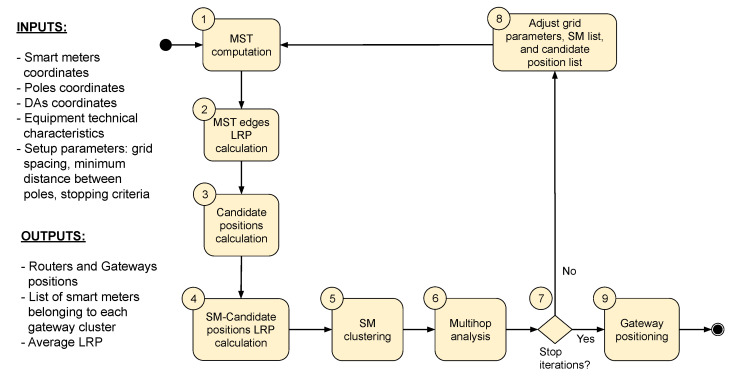
AIDA method.

AIDA is an iterative method that aims to minimize the number of unconnected SMs at every execution. A set of unconnected SMs is submitted to the method to be processed at each iteration. After the current iteration is finished, the percentage of unconnected SMs ($P_{u}$) is evaluated. A new iteration starts if this percentage is greater than the stopping criteria ($P_{u}^{max}$), which establishes the maximum of unconnected SMs accepted by the simulation. A list of remaining unconnected SMs is processed in the subsequent run. The AIDA method includes the following steps (Figure 2):

#### 4.4.1. Step 1—MST Computation

In this step, a single minimum spanning tree (MST) based on SMs geographic coordinates is computed by an Euclidean minimum spanning tree (EMST) dual-tree Boruvka algorithm [40]. The MST computation considers all the unconnected smart meters of the iteration.

The goal of using an MST formed by SM coordinates (SM-based MST) is to obtain the shortest path between a meter and its nearest neighbors. We can identify feasible communication paths between these elements from the virtual connections obtained by the MST. Doing so minimizes the number of analyses between each meter and the meters around it, as it limits the analysis to the smart meters connected by the MST edges. This strategy is useful for applying the method in large-scale scenarios.

#### 4.4.2. Step 2—MST Edges LRP Calculation

**Figure 3:**
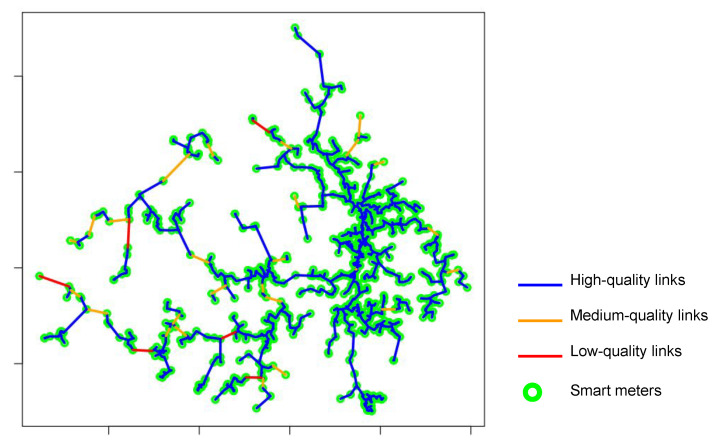
Example of MST with edges colored according to the LRP values.

When the SM-based MST is established, the LRP calculation is performed for all MST edges. Based on technical information about the devices’ reception sensitivity, three categories of LRP are established: (1) blue edges indicate high-quality links; (2) orange edges indicate medium-quality links with uncertain connection possibility; (3) edges are classified as red, indicating low-quality links, as they do not present conditions for connection. Thresholds for high, medium and low-quality links are specified in Section 5. As shown in Figure 3, the coloring of the edges allows the creation of a heatmap that helps to identify the areas where the connection is feasible and where it is uncertain.

Equation (12) is considered for the LRP calculation. See Section 4.3 for more details.

#### 4.4.3. Step 3—Candidate Positions Calculation

The set of candidate positions (CPs) for installing key devices is established based on the positions of poles with and without DA devices. As mentioned before, the method AIDA uses a grid approach to make the optimized selection of a subset of poles to minimize the computational effort required to choose the most ideal positions for the placement of the routers and gateways. In addition, we opted for the use of a grid to establish the uniform coverage of the entire region. Usually, the territorial area of cities is irregular, presenting a varied concentration (density) of poles and meters, including very dense urban and sparse rural areas. Therefore, grid-based approaches presented good results in mesh network scenarios, such as those evaluated by the authors in [26].

**Figure 4:**
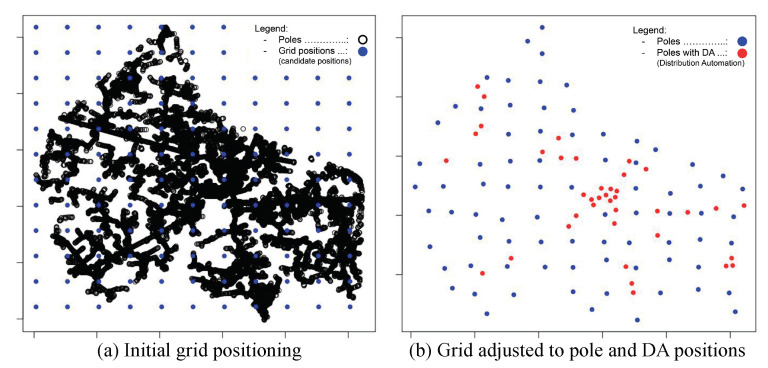
Initial grid and candidate positions placement.

First, the region area is divided into a grid with the same horizontal and vertical spacing, considering a theoretical transmission range for the routers/gateways devices. Once the initial grid was established, the grid points are adjusted to the nearest positions of poles available in the region (Figure 4), prioritizing the use of poles with DA (objective function (3)).

The method tries to keep the grid points within a minimum separation distance to minimize the allocation of candidate positions. Grid points that are too far from the poles are discarded. The grid resolution is adjusted at each AIDA iteration to decrease the distance between the points. Poles selected to be CP positions in previous iterations are ignored.

#### 4.4.4. Step 4—SM-Candidate Positions LRP Calculation

This step calculates the LRP values between each smart meter and the closest candidate positions within a specific pre-established range.

Considering that the number of smart meters is usually high and knowing that the candidate positions established for each iteration are dispersed throughout the region, the AIDA method seeks to minimize the amount of LRP calculations between meters and CPs. For this, it establishes a theoretical range and calculates the received power only for the positions within that radius.

Initially, to identify the CPs within the meter range, the distances between each meter and all the CPs of the iteration are calculated. After that, the LRP is calculated and stored in an auxiliary structure only for the relationships in which the distance between the meter and the CP is smaller than $c_{j}^{r}$ (which corresponds to the communication radius established for the candidate positions). Table 2 describes $c_{j}^{r}$ and Table 3 presents the gateway/router communication range considered by the method for the experiments.

In this step, only the LRP values are calculated for all the possible connections. However, no capacity check is performed regarding the number of connections to the CPs. The capacity check is performed by the clustering processes defined in Step 5—SM Clustering (Section 4.4.5). For the LRP calculation, Equation (12) is considered. See Section 4.3 for more details.

#### 4.4.5. Step 5—SM Clustering

This step refers to grouping SMs to a particular candidate position. In this process, LRP values calculated in the previous step are evaluated, as are the maximum accepted connections of the CPs (the maximum amount of SMs that can be connected to a CP). A connection can be established if the minimum LRP ($l_{min}$) is computed for an SM⇔CP link.

Two heuristic approaches are evaluated for clustering: bottom–up and top–down. At each iteration of AIDA, both approaches are executed, and the one that results in the smallest number of CPs will be taken as the basis for defining the list of SMs and poles for the next iteration. Both approaches aimed to reach the objectives expressed by the objective functions (1), (2), (4) and (5).

The clustering approaches are described as follows (see Table 2 for their input and output parameters):

- Bottom–Up Approach (BU): In this approach (Algorithm 1), an exhaustive search strategy is used to evaluate the LRP values calculated for the link between each smart meter and the CPs in their range. An SM⇔CP connection is established with the position with the highest LRP value. This aims to maximize the LRP value between the smart meter and the router/gateway to which it will be connected.
- Top–Down Approach (TD): In this approach (Algorithm 2), a greedy search strategy is used to connect the maximum number of SMs to each CP, presenting $LRP\geq l_{min}$, prioritizing the connection to the SMs with higher values of received power. This aims to maximize the use of the CP, connecting to it as many meters as possible, limited to $n_{max}$ (see Table 2).

**Algorithm 1** Bottom–Up Approach (BU)1:**Input:** V, C, L, $l_{min}$, $n_{max}$2:**Output:**$V_{con}$3:$V_{con}\leftarrow \left\{\right\}$4:**for all** $v_{i}\in V$**do**5:    K← Select all $c_{j}\in \{C$|$\left(c_{j}^{n}<n_{max}\right)\wedge \left(dist\left(v_{i},c_{j}\right)\leq c_{j}^{r}\right)\}$6:    $l_{max}\leftarrow l_{min}$7:    $c_{s}\leftarrow \left\{\right\}$8:    **for all** $c_{k}\in K$ **do**9:        $l_{con}\leftarrow \{l_{v_{i},c_{k}}$|$\left(l_{v_{i},c_{k}}\in L)\right\}$10:        **if** $l_{con}\geq l_{max}$ **then**11:           $l_{max}\leftarrow l_{con}$12:           $c_{s}\leftarrow c_{k}$13:        **end if**14:    **end for**15:    **if** $c_{s}\neq \left\{\right\}$ **then**16:        $V_{con}\leftarrow V_{con}\cup \left\{<v_{i},c_{s},l_{max}>\right\}$17:        $c_{s}^{n}\leftarrow c_{s}^{n}+1$18:    **end if**19:**end for**

**Algorithm 2** Top–Down Approach (TD)
1:**Input:** V, C, L, $l_{min}$, $n_{max}$2:**Output:** $V_{con}$3:M← Select all $v_{i},c_{j}\in \{L$|$l_{v_{i},c_{j}}\geq l_{min}\}$4:Q← Select distinct $c_{j}$ from M5:

$V_{con}\leftarrow \left\{\right\}$

6:**while** True **do**7:    K← Select all $c_{j},(count\left(v_{i}\right)$ as $n_{v})\in \{M$|$n_{v_{z}}>n_{v_{z+1}}\}$8:    **if** K≠{} **then**9:        $s_{i}\leftarrow$$\{k_{1}$|$k_{1}$ is the first $c_{j}$ of K}10:        N← Select ${\left(v_{i},c_{j}\right)}_{n}\in \{M$|$\left(c_{j}=s_{i}\right)\wedge \left(n\in \left\{1,2,..,n_{max}\right\}\right)\wedge \left(dist\left(v_{i},c_{j}\right)\leq s_{i}^{r}\right)\wedge ({\left(l_{v_{i},c_{j}}\right)}_{z}$>${\left(l_{v_{i},c_{j}}\right)}_{z+1})\}$11:        M←M−N12:        $Q\leftarrow Q-\{s_{i}\}$13:        $V_{con}\leftarrow V_{con}\cup \left\{<v_{i},s_{i},l_{v_{i},s_{i}}>\;\forall \;v_{i}\in N\right\}$14:    **else**15:        exit16:    **end if**17:    **if** Q={} **then**18:        exit19:    **end if**20:
**end while**



#### 4.4.6. Step 6—Multihop Analysis

The bottom–up and top–down approaches establish the one-hop feasible connection between candidate positions and smart meters. Smart meters not connected by the described approaches can then connect to a cluster of SMs using multihop connections.

The MST computed with the SMs positions is considered for the theoretical multihop connection analysis. First, smart meters belonging to SMs clusters (i.e., SMs connected to candidate positions) and their links with MST edges are identified. Then, for each SM that already belongs to a cluster, a search is conducted for adjacent MST neighbors (MST vertices) that are not yet connected but have enough received power to establish the connection (i.e., MST blue edges, with $LRP\geq l_{min}$). The search is made up to the maximum hops ($h_{max}$) limit established by the method. Neighbors with $LRP\geq l_{min}$ are connected to the SM cluster. When finding a neighbor with a received power value lower than the minimum required, such a node is discarded. It remains unconnected because there are no technical conditions for the connection.

#### 4.4.7. Step 7—Stop Iterations

For the AIDA method, a new iteration is counted for every method’s cycle execution that includes steps 1, 2, 3, 4, 5, and 6 (Figure 2). The number of iterations executed by the method depends on the number of iterations demanded to reach the expected smart meter coverage, and it will vary according to the conditions of the region, its shape, the number of poles, and the quantity and concentration of smart meters.

After each AIDA iteration, the method checks whether all the SMs are connected or whether the stopping criteria (Table 3) have been reached. If the percentage of unconnected SMs ($P_{u}$) is greater than the established ($P_{u}^{max}$), a new iteration must be performed to minimize it (objective function (6)) and the Step 8—Adjust Grid, SM and CP Lists is executed (see Section 4.4.8). Otherwise, the method may execute the gateway positioning analysis (Step 9—Gateway Positioning, see Section 4.4.9).

#### 4.4.8. Step 8—Adjust Grid, SM, and CP Lists

AIDA is an iterative method and, after the execution of an iteration, if the established percentage of unconnected SMs ($P_{u}^{max}$) has not yet been reached, some parameters must be adjusted before starting another method execution.

The parameters to be considered in a new iteration include the list of SMs that remained unconnected in the previous iteration and the list of CPs used. Regarding the calculation of the CPs for the new iteration, the grid used to position the candidate positions must be adjusted, usually adopting a new horizontal and vertical space between the grid points that represents half of the value used in the previous iteration, resulting in a denser grid (as indicated in Table 4). The information about the CPs used in the previous iterations is important because, for the new iteration, the positions of poles previously considered as CPs are ignored in new iterations.

#### 4.4.9. Step 9—Gateway Positioning

In previous steps from the method, AIDA identified the best pole positions for installing routers and gateways. Initially, pole positions are selected with the help of a grid and promoted to candidate positions. From these candidate positions, some positions are selected as the most suitable for the positioning of key devices (i.e., routers and gateways). This selection of CPs is made in Step 5—SM Clustering of the AIDA method (Section 4.4.5). The CPs considered in the gateway positioning will be the ones computed by the SM clustering approach (bottom–up or top–down) that effectively minimizes the number of required CPs.

**Figure 5:**
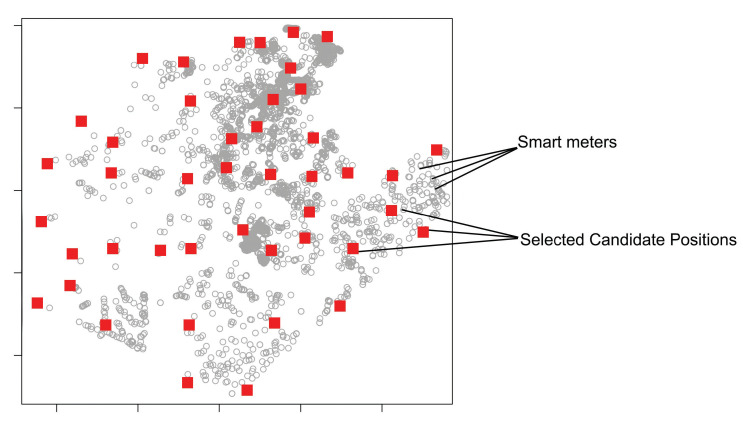
Selected Candidate Positions.

In the gateway positioning step, described in this section, the candidate positions selected for a region (see example in Figure 5) are evaluated to determine whether they will be considered as positions for installing routers or gateways. In principle, all selected candidate positions are considered valid positions for router placement.

**Figure 6:**
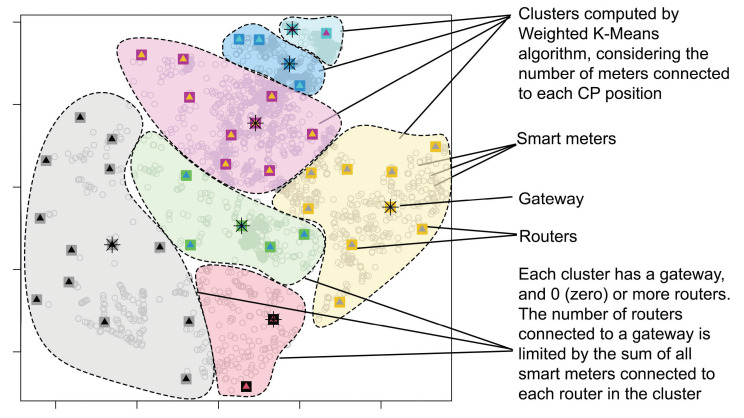
Gateway positioning. The figure presents the different clusters computed by the Weighted K-Means algorithm. Each group has a gateway positioned and can have zero or more routers.

Subsequently, a clustering process is used to determine the set of routers that will be connected to the same gateway. For this, the weighted K-means algorithm [41] is used to select the list of routers that have to be included in the same cluster and to establish the best position for the gateway on each cluster. Figure 6 illustrates the result of the clustering process of this step. The input parameters of this clustering algorithm include the list of selected CPs (already considered router positions), the number of meters connected to each candidate position, and the maximum connections accepted by a gateway. The number of meters connected to each CP defines the weight of each CP. The clustering process aims to group to the same cluster a list of the closest CPs whose sum of meters connected to each one does not exceed the limit of connections established for the gateway. This limit is a parameter determined for the execution of the method (varying according to the technical characteristics of the equipment).

In some situations, it may occur that a given group computed by the weighted K-Means algorithm only contains one selected candidate position. This may happen because the number of meters connected to the CP is equal to or near the limit of meters established for the cluster. In this case, this position will be classified as a gateway. In other cases, the group will be formed by a set of routers and a gateway (selected among the positions of CPs in the cluster).

After this clustering process, the positions of the key devices are established, and the AIDA method is concluded.

### 4.5. Computational Complexity

For the computational complexity analysis, more specifically, for the Time Complexity analysis of the AIDA method, it is necessary to consider its main components, which include the dual-tree Boruvka minimum spanning tree (Euclidean MST—EMST) algorithm, the bottom–up (BU) and the top–down (TD) clustering approaches, the depth-first search (DFS) algorithm to establish the MST multihop analysis, and the weighted K-means algorithm, used for grouping routers and selecting gateway positions.

The MST, the clustering algorithms (BU and TD), and the depth-first search algorithm can be run over several iterations until the expected connectivity coverage for existing smart meters is achieved. The weighted K-means algorithm, in turn, is executed only once as the last step of the AIDA method.

According to the authors in [40], the EMST dual-tree Boruvka algorithm presents the complexity, T(MST), established by (13):

(13) T(MST)≈O(|V|×log|V|)

where |V| corresponds to the total points of the *MST*, which is equal to the number of smart meters.

To calculate the complexity of the bottom–up (*BU*) algorithm, we can observe the existence of two nested loops (see Algorithm 1). The time complexity must be calculated considering the number of meters |V|, which establishes the number of executions of the main loop, the existence of the select code responsible for filtering candidate positions that are within reach of each meter, and the execution of the inner loop, which identifies the ideal candidate position for connecting the meter under analysis. The complexity of the BU algorithm, T(BU), can be established by Equation (14):

(14) $$\begin{array}{cc} T(BU) & =O(|V|\times |C|) \end{array}$$

where |V| is equal to the number of meters and |C| is equal to the number of candidate positions within the range of each SM, in the worst case.

For the top–down (*TD*) algorithm (see Algorithm 2), we can calculate the complexity by observing the existence of one loop that uses a select instruction at each execution to identify/update the list of CPs available for processing. In addition, another select function is performed to update the list of possible connections between meters and CPs. Based on this description, the complexity of the TD algorithm, T(TD), can be established by Equation (15):

(15) $$\begin{array}{cc} T(TD) & =O(|C|\times n_{max}) \end{array}$$

where |C| corresponds to the number of CPs (considered as the worst case), and $n_{max}$ indicates the maximum number of SMs per CP.

For the depth-first search algorithm used for the multihop analysis, the complexity T(DFS) is computed as (16):

(16) T(DFS)=O(|V|+|E|)

where |V| is the number of nodes (smart meters) and |E| is the number of edges (connections between smart meters) of the MST.

The complexity of the weighted K-means algorithm, T(WK), is estimated with Equation (17):

(17) $$T\left(WK\right)=O(|C{|}^{2})$$

where |C| is equal to the number of candidate positions selected to be grouped.

Considering the need for a small number of *i* iterations, the complexity of the *AIDA* method (T(AIDA)) can be established as (18):

(18) $$\begin{array}{cc} T(AIDA) & =O(i\times \max\{T(MST),T(BU),T(TD),T(DFS)\}+T(WK))\\ \; & =O(i\times \max\{N\times \log N,|V|\times |C|,|C|\times n_{max},|V|+\left|E|\right\}+{\left|C\right|}^{2})\\ \; & =O(\max\{|V|\times \log|V|,|V|\times \left|C|,|C\right|\times n_{max},|V|+\left|E|\}\right)\\ \; & =O(|V|\times |C|) \end{array}$$

The worst-case analysis of the method is very pessimistic, as it assumes that all smart meters can reach all candidate positions. In practice, this situation is improbable since the communication range of meters and routers is quite limited. However, an average-case analysis is unfeasible due to the diversity of meter geographic concentration, terrain profiles, the number of candidate positions, and aspects of communication devices that change a lot from one scenario to another.

## 5. Experiments and Results

The experiments carried out in this study include real data from four regions in the state of Paraná, located in the south of Brazil. The regions are urban areas with a large concentration of smart meters and rural areas with dispersed smart meters. The four selected regions were named Region A, Region B, Region C, and Region D. Table 5 presents details about these regions, including the total number of SMs, bounding box (BB) area, SM density per km${}^{2}$, number of poles, and number of distribution automation (DA) devices installed.

Experiments were conducted using the parameters detailed in Table 3 that include real equipment data (e.g., operating frequency, transmission power, antennas gains, and height) and some quality criteria to be observed during the method execution, such as the minimum LRP value to establish a connection, the maximum number of hops, and the stopping criteria.

The parameters presented in Table 4 were considered to establish the candidate positions for each iteration of the method (Steps 3 and 8, described in Section 4.4.3 and Section 4.4.8).

Two different experiments were executed using the AIDA method:

- Case Study 1: Experiments with AIDA, using its link-specific propagation model;
- Case Study 2: Experiments with AIDA, using Erceg-SUI propagation model.

The experiments and results are presented as follows.

### 5.1. Case Study 1: Experiments with AIDA, Using Link-Specific Propagation Model

In this section, we describe the experiments with the AIDA method using its original propagation model, which calculates losses considering the detailed terrain profile between the communication devices in the analysis.

**Figure 7:**
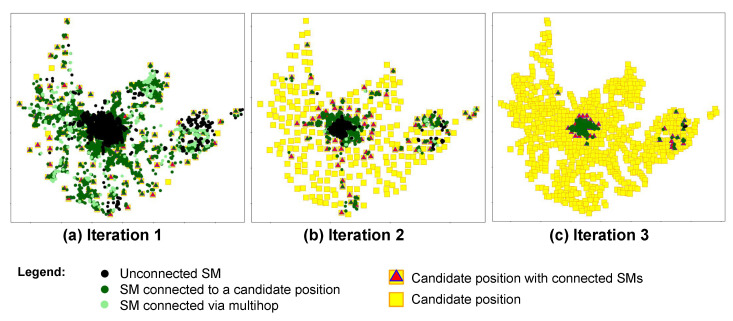
AIDA iterations for Region A.

Figure 7 shows the result after each process iteration in region A (150,951 SMs). It can be noticed that, at the end of each iteration (iterations 1, 2, and 3), the number of unconnected SMs (points in black) decreases. The green points show the SMs connected to the CP’s positions via one-hop or multihop connections. The figure also presents the candidate positions available and the ones effectively selected to connect the SMs.

A summary of the regions A, B, C, and D results is presented in Table 6. For each iteration, AIDA performs the processing with two approaches (bottom–up and top–down) using the same set of SMs, i.e., the list of unconnected SMs resulting from the previous processed iteration with the approach that used the smallest number of CPs.

Regarding the main characteristics of the two different approaches, it can be said that: the BU approach prioritizes the connection of a smart meter with the candidate position that presented higher LRP values, resulting in a final network topology that tends to present a higher average LRP value when compared to the other approach; the TD approach, in turn, prioritizes the use of fewer candidate positions than the BU approach, respecting the minimum LRP value to establish the connections between candidate positions and smart meters around them, reaching a final average LRP value slightly smaller than the one obtained by the other approach.

Considering this, for the analyzed regions, the top–down approach presented better results overall, especially regarding the number of CPs selected.

By evaluating the number of *CPs* demanded by each clustering approach (Table 6), we notice that the top–down approach can reduce the number of selected key devices for all the regions. Equation (19) defines the relative gain between the top–down (*TD*) and the bottom–up *(BU)* approach regarding the reduction in the number of CPs.

(19) $$G_{r}\left(\%\right)=\left(1-\frac{CP_{s}^{TD}}{CP_{s}^{BU}}\right)\cdot 100$$

Despite the bottom–up approach presenting (for all four regions) a better average LRP for the links than the top–down approach, the gains in the number of CPs effectively selected to install routers and gateways reflect cost minimization. In addition, the top–down approach assures average LRP values that qualify the links in a high-quality (with $LRP\geq l_{min}$) threshold. Considering the gains in the number of CPs (15.3% for Region A, 24.1% for Region B, 11.1% for Region C, and 8.3% for Region D), the average reduction in the number of CPs (comparative gain) is 14.7%.

Regarding the number of CPs computed by the top-down approach, Region A demanded 249 positions. Regions B and D, in turn, resulted in the same number of candidate positions used (44 positions), despite having very different amounts of SMs (56,157 SMs for Region B and 6106 for Region D). This behavior is justified because Region B has a higher concentration of SMs per km^2^. In contrast, region D has the geographically dispersed meters, requiring the positioning of various key devices to ensure communication. For Region C, a total of 169 devices was estimated as it is also a region with high sparsity (7.2 SMs/km^2^).

Analyzing the first iteration of the method, the number of candidate positions with which a meter has viable conditions for establishing a connection varied (on average) from 1.9 possibilities for Region D to 6.2 possibilities (on average) for each SM in Region B. For Region A, it was observed that an SM could establish a connection with up to 15 candidate positions.

### 5.2. Case Study 2: Experiments with AIDA, Using Erceg-SUI Propagation Model

In this section, we describe the additional experiments carried out to evaluate the AIDA method using a general path loss model, which does not assess the particularities of the terrain profile between specific smart meters and candidate positions.

For this, the method AIDA was adapted to use the Erceg-SUI propagation model (SUI model), implemented as presented by the authors in [6,7] (IEEE 802.16.3c-01/29r4, section Suburban Path Loss model). The Erceg-SUI model corresponds to a path loss model computed by considering a general classification of the terrain in which the communication devices are installed.

The Erceg-SUI model considers the existence of the following terrain types: Category A—hilly/moderate-to-heavy tree density; Category B—hilly/light tree density or flat/moderate-to-heavy tree density; Category C—flat/light tree density. Originally, the model was specified based on data collection in 95 regions of the United States considering a transmission frequency of 1.9 GHz. Thus, for its use in our experiments, frequency and receiver antenna height correction factors were used as recommended in [7].

According to [6,7], the equation for calculating the Erceg-SUI model Path Loss is expressed as (20):

(20) $$PL_{SUI}=A+10\gamma \log_{10}\left(d/d_{0_{SUI}}\right)+s;\;\;\;d\geq d_{0_{SUI}}$$

where *A* is the decibel path loss at distance $d_{0_{SUI}}$ and *s* is the shadow fading variation about the linear relationship. The authors in [6] call *A* the intercept value, and they choose a value for $d_{0_{SUI}}$ of 100 m. In addition, $A=20\;\log_{10}\left(4\;\pi \;d_{0_{SUI}}/\lambda \right)$, where λ is the wavelength in m, γ is the path-loss exponent with $\gamma =\left(a-bh_{b}+c/h_{b}\right)+x\sigma_{\gamma }$ for $h_{b}$ between 10 m and 80 m ($h_{b}$ is the height of the base station in m), and *a*, *b*, and *c* are constants dependent on the terrain category, as shown in Table 7. The shadowing effect (*s*) follows a lognormal distribution, and it is calculated using (21):

(21) $$s=10\;x\;\sigma_{\gamma }\;\log_{10}\left(d/d_{0_{SUI}}\right)+y\;\mu_{\sigma }+y\;z\;\sigma_{\sigma }$$

where *x*, *y*, and *z* are independent zero-mean Gaussian variables of unit standard deviation, N[0,1], as defined in [6]. The frequency and receiver antenna height correction factors are calculated by Equations (22)–(24). Equation (22) defines the frequency correction factor, where *f* is the frequency in MHz.

(22) $$\Delta PL_{f}=6\;\log_{10}\left(f/2000\right)$$

The $\Delta PL_{h}$ (in dB) is the receiver antenna height correction term given by Equations (23) and (24), where *h* is the height of the antenna, between 2 m and 10 m.

(23) $$\Delta PL_{h}=-10.8\;\log_{10}\left(h/2\right);\;\;\mathrm{for}\mathrm{Categories}A\mathrm{and}B$$

(24) $$\Delta PL_{h}=-20\;\log_{10}\left(h/2\right);\;\;\mathrm{for}\mathrm{Category}C$$

The path loss with the application of correction factors is calculated as follows (25):

(25) $$PL_{modified}=PL_{SUI}+\Delta PL_{f}+\Delta PL_{h}$$

For case study 2 experiments, the AIDA method was adapted to use the modified path loss presented by the Erceg-SUI model instead of the Delta-Bullington model. Experiments were carried out using the top–down approach of the AIDA method, considering the four regions previously used and performing an experiment for each region (A, B, C, D) and each type of terrain (category A, category B, and category C) proposed by the SUI-model.

**Figure 8:**
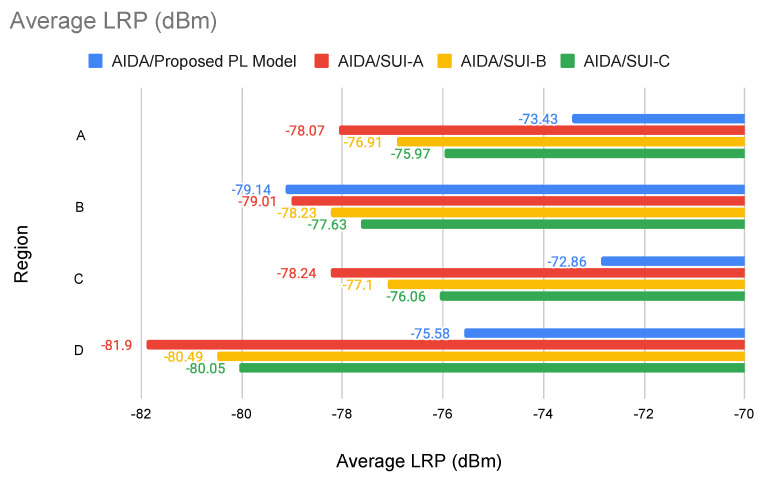
Average LRP values (in dBm) obtained by the AIDA method using its original path loss model (AIDA/proposed PL model) compared to the values obtained by the AIDA method using Erceg-SUI path loss model for the terrain Category A (AIDA/SUI–A), Category B (AIDA/SUI–B), and Category C (AIDA/SUI–C). The chart compares the performance of AIDA for the Regions A, B, C, and D, used in the experiments.

The results presented in Table 8 show that, in most cases, using the AIDA method, it was possible to obtain a better average LRP value than that obtained with the SUI-model (Figure 8). By evaluating the terrain in a more detailed way, it can even be said that the particularities of the terrain between the devices are more faithfully evaluated, leading to an LRP calculation that tends to be more accurate. An exception is Region B, which presented better results for the Erceg-SUI path loss model. Region B has the highest smart meters concentration per km${}^{2}$ (316.9 SMs/km${}^{2}$), where most of them are very close to each other.

**Figure 9:**
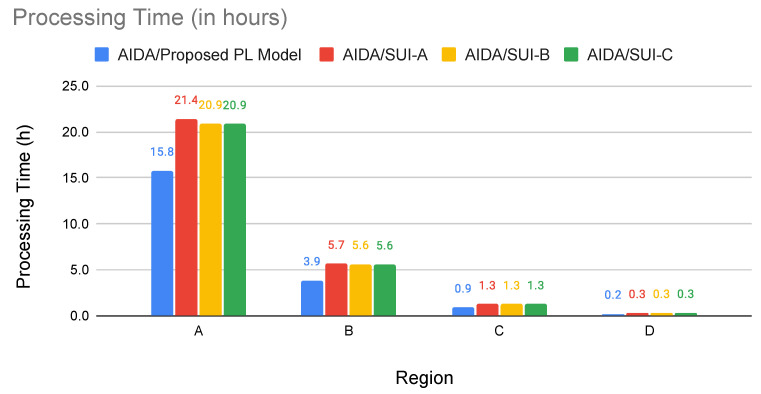
Processing time (in hours) consumed for the execution of the AIDA method using its original path loss model (AIDA/proposed PL model) compared to the values demanded by the AIDA method using the Erceg-SUI path loss model for the terrain Category A (AIDA/SUI–A), Category B (AIDA/SUI–B), and Category C (AIDA/SUI–C). The chart compares the performance of AIDA for the Regions A, B, C, and D, used in the experiments.

Tests with the SUI-model path loss resulted in more unconnected smart meters between each AIDA iteration, generating the need to evaluate more connection possibilities and demanding more processing time (Figure 9).

**Figure 10:**
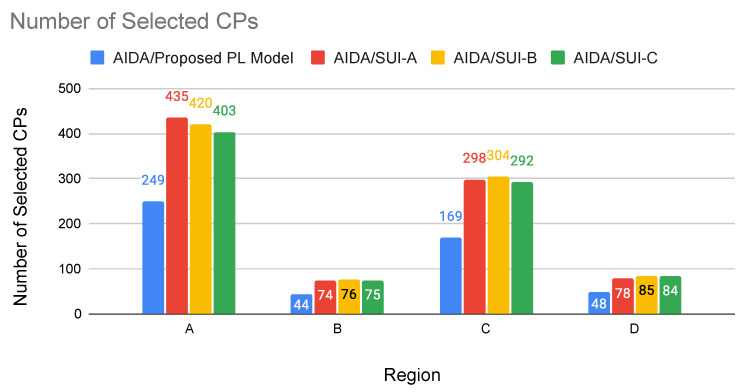
Number of CPs selected by the AIDA method using its original path loss model (AIDA/proposed PL model) compared to the values selected by the AIDA method using the Erceg-SUI path loss model for the terrain Category A (AIDA/SUI–A), Category B (AIDA/SUI–B), and Category C (AIDA/SUI–C). The chart compares the performance of AIDA for the Regions A, B, C, and D, used in the experiments.

Using the AIDA method with its original propagation model (i.e., taking into account the terrain profile), it was possible to observe a smaller number of candidate positions selected for the installation of routers, suggesting that the topology computed by the AIDA method with the original path loss/diffraction model can plan the smart meter’s coverage with a lower number of routers (i.e., selected candidate positions, as shown by Figure 10) and (in most cases) higher average LRP value, resulting in a more efficient solution.

By evaluating the number of CPs demanded by AIDA (Table 8, column AIDA (TD approach)), we can notice that the method presented in this study can reduce the number of selected CPs for all the regions when compared with the values calculated by the approaches using Erceg-SUI path loss model. Equation (26) defines the average relative gain between AIDA (executed using its link-specific propagation model) compared to the use of AIDA executed using the Erceg-SUI propagation model. The gain is calculated regarding the number of CPs selected by AIDA executed using each different propagation model. It aims to show the reduction in the number of candidate positions demanded to connect the smart meters of the region when the original AIDA method is used.

(26) $$G_{AIDA}\left(\%\right)=\left[1-\left(\frac{1}{3}\sum_{t=A}^{C}\frac{CPs^{AIDA}}{CPs^{SUI_{t}}}\right)\right]\cdot 100$$

Using AIDA, the average relative gain regarding the number of selected CPs varied from 40.562% for Region A to 43.273% for Region C.

**Figure 11:**
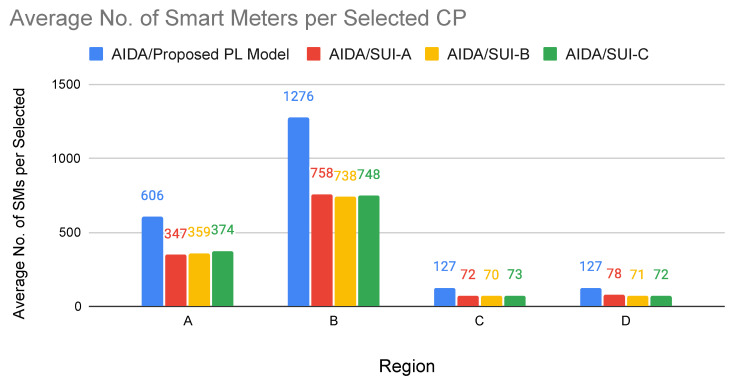
Average number of smart meters (SMs) connected to each CP selected by the AIDA method using its original path loss model (AIDA/proposed PL model) compared to the values connected to the CPs by the AIDA method using Erceg-SUI path loss model for the terrain Category A (AIDA/SUI–A), Category B (AIDA/SUI–B) and Category C (AIDA/SUI–C). The chart compares the performance of AIDA for the Regions A, B, C, and D, used in the experiments.

In addition, it can be noticed (Figure 11) that the average number of smart meters per selected CP is higher for the AIDA method. This suggests that AIDA can better utilize the connection capacity of a candidate position as it demands a lower final amount of installed routers/gateways compared to using the method with a general path loss model.

## 6. Discussions and Future Work

In this section, we review the main objectives of the work and evaluate the results obtained from the research.

The main research objectives include the following:

- The presentation of an efficient AMI planning method with a large-scale focus.
- The use of a propagation model that does not depend on an empirical terrain classification.
- The use of a heuristic approach based on a spanning tree and clustering, capable of evaluating a smaller number of connections and resulting in topologies that use fewer routers and gateways.

Regarding the applicability of the method in large-scale scenarios, experimentation with real data from regions of Brazil totaling more than 230,000 smart meters demonstrated that the proposed method effectively deals with datasets from regions with different degrees of density of smart meters and poles, including high-density regions (such as dense urban regions) and regions with high sparseness (rural regions or industrial areas). As observed in Table 6, the processing times for the different real regions considered consumed from 14 min 19 s (for Region D) to 15 h 47 min 44 s (for Region A).

The use of a path loss model that calculates losses considering the particularities of the terrain between the devices permitted the evaluation of the possibility of connection more precisely, with the advantage of visualizing the connectivity condition for the entire region (as can be seen with Figure 3). The average LRP values obtained in the processing of each region evaluated (varying from −79.14 dBm for Region B to −72.86 dBm for Region C, according to Table 6) show that the use of a detailed path loss model made it possible to achieve an optimized result.

The implemented heuristic approach demonstrated efficiency for the different scenarios evaluated as the number of candidate positions calculated for each region was enough for the connection of the smart meters respecting the value of LRP established for the experiments.

Using a grid strategy to select poles helps minimize the final number of installation positions and optimizes the method’s performance by decreasing the number of connections to be evaluated. For example, in Table 5, it is possible to observe that Region A has 62,412 poles. Despite this, we observed (Table 6) that the total number of poles selected for region A (for the installation of routers and gateways) is only 249 poles (using the top–down clustering approach). It is important to highlight that, for Region A, the process of candidate position calculation establishes 1245 poles to be evaluated (163 poles for the first iteration, 289 poles for the second iteration, and 793 poles for the third iteration of the method). Despite the region having 62,412 poles, only 1245 pole positions (1.995%) were evaluated to establish the connectivity of the meters in the region. This type of simplification reinforces the method’s applicability for large-scale scenarios since there is a significant decrease in the number of calculations performed with the implemented strategy.

The multihop analysis for connecting meters using a minimum spanning tree algorithm also helps reduce the complexity of the problem and reduce the calculations to be performed, as only a subset of possible connections between smart meters needs to be evaluated.

Regarding scalability, it is possible to highlight that the method worked well regarding the processing time, reaching shorter execution times when compared to usage scenarios in which it was considered a simplified path loss model. In fact, the results in Table 8 demonstrate that with its original path loss model, AIDA presents a better processing time than AIDA using the Erceg-SUI model that does not demand a detailed analysis of the terrain characteristics.

Even with the results representing satisfactory values, it is important to highlight that the method can be improved to reduce the processing time since processing bottlenecks were identified and deserve improvements in future method updates. For example, for a region with 2375 smart meters used in a special experiment for execution profile analysis, from a total processing time of 244.0 s, the time consumed for the main processes was as follows (Table 9): multihop connection analysis, 11.2 s; clustering with a bottom–up approach, 4.4 s; clustering with top–down approach, 1.6 s. However, loading, selecting, grouping, filtering, and saving datasets summed 169.5 s, especially due to the high volume of disk I/O operations involved. The remaining 57.3 s of the required processing time were consumed by smaller processes, such as calls to external functions (necessary to obtain topographic profile data) and other processes inherent to the execution platform used.

In summary, the results obtained with the AIDA method proposed by this study indicate that the experiments carried out (considering case studies 1 and 2) were able to obtain results with coverage and connection values within established values for all evaluated regions. Even when comparing the AIDA method with its original loss model and the AIDA method with a path loss model that does not use a detailed terrain analysis, the results obtained (especially concerning the number of CPs selected) were better. Additional experiments can be performed after the bottleneck mitigation and future modifications expected for the path loss model used by the method.

Future work will consider the improvement of the link propagation model, including the existing vegetation model, as well as the type of environment (e.g., urban, dense urban, rural area). Additional experiments may explore the use of AIDA in expanding and optimizing already installed smart grid communication networks.

For rural or sparse regions with less DA equipment or no backhaul infrastructure coverage, there may be a need to assess the network expansion to ensure integration between the AMI and Backhaul networks. However, as these are particular situations, they are not part of the scope of the current study and are reserved for future works.

## 7. Conclusions

This study presented the AIDA method for router/gateway placement in AMI planning that evaluates the LRP between SMs and key device positions, calculated based on a detailed analysis of the terrain profile. In addition, the method presented the use of an MST-based technique for the multihop connectivity analysis aiming to minimize the number of connections to be analyzed.

The research explored using AI-driven heuristic approaches and clustering techniques for the method implementation. To investigate the method’s capacity to deal with large-scale smart grids, the experiments evaluated real data from cities in the south of Brazil totaling over 230,000 smart meters. Experiments in four large-scale scenarios demonstrate that the coverage and average LRP values computed by the AIDA method are within established connection thresholds, suggesting topologies that consider the actual terrain profile particularities and other system requirements.

Using a detailed terrain profile analysis to compute the path loss between devices to be connected demonstrated viability even for the largest region evaluated (with over 150,000 smart meters). The AIDA method used two optimized strategies to minimize the number of connections to be evaluated. First, it used a grid approach to select candidate positions. Second, it uses an MST to evaluate the multihop connections in cases where the smart meter cannot directly connect to a router or gateway but through another smart meter.

The AIDA method introduces two different smart meters clustering approaches: bottom–up, which privileges the quality of links; and top–down, which focuses on demanding fewer devices. Furthermore, we presented the computational complexity (time complexity) of these two clustering approaches and the method’s main components descriptively to allow the reader to visualize the participation of each element in the execution time of the method.

In the experiments, the top–down approach reduced by 14.7%, on average, the total number of gateways and routers positioned compared to the bottom–up approach. Bottom–up, in turn, presented a higher average LRP for the links, prioritizing the quality of the links over cost minimization. Considering that both strategies can present results ensuring high-quality links, both are applicable and may be selected according to the smart grid scenario constraints and objectives, prioritizing the use of candidate positions with DAs.

In addition, experiments using a general path loss model (Erceg-SUI model) demonstrated that the AIDA can reach solutions demanding fewer key devices yet ensuring LRP values in the established threshold. In these experiments, AIDA used its proposed propagation model, and presented an average relative gain regarding the number of selected CPs varying from 40.562% to 43.273% when compared to AIDA using the Erceg-SUI model as the propagation model. Furthermore, regarding the processing time, the AIDA method with its original path loss model proved to be more efficient than when evaluated using the general path loss model.

## Figures and Tables

**Table 2:** Input and output parameters of the algorithms.

Parameter	Description
$V=\{v_{1},\ldots ,v_{u}\}$	Set of smart meters, $v_{i}$
$C=\{c_{1},\ldots ,c_{z}\}$	Set of CPs, $c_{j}$, where $c_{j}=<c_{j}^{n},c_{j}^{r}>$
$c_{j}^{n}$	Number of SMs connected to $c_{j}$
$c_{j}^{r}$	Communication range of $c_{j}$
L	Set of LRP values, $l_{v_{i},c_{j}}$, for links $v_{i}↔c_{j}$
$V_{con}$	Set of SMs connected to CPs, $<v_{i},c_{j},l_{v_{i},c_{j}}^{max}>$
M, N	Subsets of L
Q	Subset of C
$l_{min}$	Minimum LRP value to establish a connection
$n_{max}$	Maximum number of SMs per CP device
$dist(v_{i},c_{j})$	Distance between SM $v_{i}$ and CP $c_{j}$
$c_{s}$	CP selected to connect an SM $v_{i}$
$n_{v}$	Counter of $v_{i}$ in the range of a CP $c_{j}$

**Table 3:** Equipment and method parameters.

Parameter	Description
Wi-SUN operating frequency	920 MHz
Smart meter transmission power ($P_{tx}$)	26 dBm
Smart meter antenna gain ($G_{tx}$)	2 dBi
Smart meter antenna height	1.5 m
Gateway/router antenna gain ($G_{rx}$)	6.25 dBi
Gateway/router antenna height	7 m
Gateway/router communication range	3000 m
Gateway/router maximum connections ($n_{max}$)	2000
Minimum distance between Poles and DA Poles	1000 m
Minimum LRP to establish a connection ($l_{min}$)	−95 dBm
Stopping criteria	$P_{u}^{max}=$ 2%
Maximum number of hops ($h_{max}$)	7
High-quality (HQ) link criteria	LRP ≥ −95 dBm
Medium-quality (MQ) link criteria	−105 ≤ LRP < −95 dBm
Low-quality (LQ) link criteria	LRP < −105 dBm

**Table 4:** Iteration parameters for grid and poles separation.

Parameter	Iter. 1	Iter. 2	Iter. 3
Grid spacing (km)	5.0	2.5	1.25
Minimum distance between poles (km)	3.0	1.5	0.75
Minimum distance between poles and poles with DA (km)	1.0	1.0	1.0

**Table 5:** Information about the regions used in the experiments.

Region	No. of SMs	BB Area (km^2^)	SMs/km^2^	Poles	DAs
A	150,951	4427.2	34.1	62,412	80
B	56,157	177.2	316.9	15,754	19
C	21,583	3001.8	7.2	26,005	39
D	6106	622.6	9.8	8250	10

**Table 6:** Final results comparing bottom–up (BU) and top–down (TD) approaches.

	Region A		Region B		Region C		Region D	
**Metric**	**BU**	**TD**	**BU**	**TD**	**BU**	**TD**	**BU**	**TD**
No. of iterations	3	3	3	3	2	2	2	2
No. of unconnected SMs	3	3	0	0	0	0	4	4
Percentage of connected SMs	99.998%	99.998%	100.0%	100.0%	100.0%	100.0%	99.934%	99.934%
No. of CPs selected for positioning gateways and routers	294	249	58	44	190	169	48	44
No. of gateways	**	140	**	44	**	18	**	7
No. of routers	**	109	**	0	**	151	**	37
Average No. of SMs/gateway	**	1078.2	**	1276.3	**	1199.1	**	871.7
No. of SMs/CP	513.4	606.2	968.2	1276.3	113.6	127.7	127.1	138.7
Average LRP for the links SM⇔SM and SM⇔CP (dBm)	−72.15	−73.43	−73.72	−79.14	−67.6	−72.86	−71.93	−75.57
% of high-quality links	99.883%		99.998%		99.407%		99.361%	
% of medium-quality links	0.081%		0.002%		0.459%		0.573%	
% of low-quality links	0.036%		0%		0.134%		0.066%	
Max. No. of CPs in SM range *	15		12		10		5	
Average No. of CPs in SM range *	5.8		6.2		4.3		1.9	
Processing time (h:min:s)	15:47:44		03:52:07		00:55:04		00:14:19	
Relative gain ($G_{r}$)	15.3%		24.1%		11.1%		8.3%	

* For the 1st iteration, considering only high-quality links; ** Computed only for the approach that selected less CPs positions.

**Table 7:** Erceg-SUI model parameters.

Model Parameter	Category A Terrain	Category B Terrain	Category C Terrain
*a*	4.6	4.0	3.6
*b* (in m${}^{-1}$)	0.0075	0.0065	0.0050
*c* (in m)	12.6	17.1	20.0
$\sigma_{\gamma }$	0.57	0.75	0.59
$\mu_{\sigma }$	10.6	9.6	8.2
$\sigma_{\sigma }$	2.3	3.0	1.6

**Table 8:** AIDA (TD approach, with its proposed PL model) results compared to the results of AIDA using the Erceg-SUI path loss model.

			AIDA method using Erceg-SUI Path Loss Model		
**Region**	**Metric**	**AIDA (TD Approach)**	**Category A Terrain**	**Category B Terrain**	**Category C Terrain**
	No. of iterations	3	3	3	3
	No. of selected CPs	249	435	420	403
	No. of unconnected SMs	3	30	17	11
A	Percentage of connected SMs	99.998%	99.980%	99.989%	99.993%
	Average LRP (dBm)	−73.43	−78.07	−76.91	−75.97
	Processing time	15 h 47 min 44 s	21 h 25 min 45 s	20 h 53 min 23 s	20 h 51 min 25 s
	AIDA Gain ($G_{AIDA}$)	40.562%			
	No. of iterations	3	3	3	3
	No. of selected CPs	44	74	76	75
	No. of unconnected SMs	0	0	0	0
B	Percentage of connected SMs	100%	100%	100%	100%
	Average LRP (dBm)	−79.14	−79.01	−78.23	−77.63
	Processing time	3 h 52 min 7 s	5 h 39 min 50 s	5 h 38 min 30 s	5 h 36 min 8 s
	AIDA gain ($G_{AIDA}$)	41.326%			
	No. of iterations	2	2	2	2
	No. of selected CPs	169	298	304	292
	No. of unconnected SMs	0	64	53	39
C	Percentage of connected SMs	100%	99.704%	99.754%	99.819%
	Average LRP (dBm)	−72.86	−78.24	−77.10	−76.06
	Processing time	0 h 55 min 4 s	1 h 15 min 17 s	1 h 15 min 9 s	1 h 15 min 40 s
	AIDA gain ($G_{AIDA}$)	43.273%			
	No. of iterations	2	2	2	2
	No. of selected CPs	48	78	85	84
	No. of unconnected SMs	4	16	18	12
D	Percentage of connected SMs	99.934%	99.738%	99.705%	99.803%
	Average LRP (dBm)	−75.58	−81.90	−80.49	−80.05
	Processing time	0 h 14 min 19 s	0 h 18 min 38 s	0 h 19 min 22 s	0 h 19 min 38 s
	AIDA gain ($G_{AIDA}$)	41.616%			

**Table 9:** Processing time (execution profile analysis) for a special experiment using 2375 smart meters.

Process	Execution Time (s)
Multihop connection analysis	11.2 s
Clustering with bottom–up approach	4.4 s
Clustering with top–down approach	1.6 s
Dataset load, select, filter, save processes	169.5 s
Miscellaneous	57.3 s
Total:	244.0 s

## Data Availability

Not applicable.

## Abbreviations

The following abbreviations are used in this manuscript:

AMI	Advanced Metering Infrastructure
BB	Bounding box
BU	Bottom–Up approach
CC	Control Center
CP	Candidate Position
DA	Distribution Automation device
DL	Diffraction Loss
DNP3	Distributed Network Protocol
IEEE	Institute of Electrical and Electronics Engineers
ITU	International Telecommunication Union
LDL	Link Diffraction Loss
LPL	Link Power Loss
LRP	Link Received Power
MST	Minimum Spanning Tree
NAN	Neighborhood Area Network
PL	Path Loss
SCADA	Supervisory Control and Data Acquisition
SDR	Successful Delivery Rate
SG	Smart Grid
SM	Smart Meter
SUI	Stanford University Interim
TD	Top–Down approach
VLANs	Virtual Local Area Network
WAN	Wide-Area Network
